# A Novel Traffic Scheduling Algorithm for Multi-CQF Using Mixed Integer Programming and Variable Neighborhood Search Genetic Algorithm in Time-Sensitive Networking

**DOI:** 10.3390/s25134197

**Published:** 2025-07-05

**Authors:** Cheng Wang, Zhiquan Lin, Yuhao Zhao, Fen Hu, Zhan Huan

**Affiliations:** 1 School of Computer Science and Artificial Intelligence, Changzhou University, Changzhou 213159, China; chengwang@cczu.edu.cn (C.W.); s24150812025@smail.cczu.edu.cn (Z.L.); 2 School of Microelectronics and Control Engineering, Changzhou University, Changzhou 213159, China; s22060858026@smail.cczu.edu.cn (Y.Z.); s22060858007@smail.cczu.edu.cn (F.H.)

**Keywords:** time-sensitive network, Multi-CQF, queueing theory, mixed integer programming, variable neighborhood search, genetic algorithm

## Abstract

Time-Sensitive Networking (TSN) is an advance Ethernet paradigm designed to provide low delay, low jitter, and deterministic transmission time. The Cycling Queuing and Forwarding (CQF) mechanism is introduced in TSN as a scheduler to achieve precise communication. Multi-CQF, as an extension of CQF, supports the transmission of various traffic types by assigning different cycle lengths to each queue group. In its original form, Multi-CQF-based scheduling algorithms do not account for flow sorting, leading to increased transmission delays and reduced network efficiency as a network dynamically changes. To enhance the performance of Multi-CQF, this paper initially utilizes queuing theory to analyze and manage traffic, providing foundation solutions. Subsequently, Mixed Integer Programming (MIP) and the Variable Neighborhood Search Genetic Algorithm (VNS-GA) are employed to optimize transmission delay in small- and large-traffic TSN networks, respectively. MIP quickly seeks out the optimal scheduling solution for small-traffic TSN networks using branch-and-bound and linear programming techniques, while the VNS-GA improves efficiency and performance for large-traffic ones by continuously adjusting the search neighborhood strategy. Comparing with other existing schemes, computer simulation reveals that MIP reduces delay by approximately 13% on average in small-traffic TSN networks, while the VNS-GA achieves an average delay reduction of 7% in large-traffic ones.

## 1. Introduction

Over the past decade, the potential of the Industrial Internet of Things (IIoT) in the era of Industry 4.0 has garnered significant attention from both academia and industry. With the rapid development of the IIoT, a large number of industrial devices will be interconnected via wired or wireless modes, presenting stringent challenges for network systems regarding stream reliability and low-delay communication [1]. Time-Sensitive Networking (TSN), as a novel network technology with high real-time capability and reliability, introduces a new spectrum of application potentials for the IIoT. Its applications are extensive, spanning industrial automation, intelligent manufacturing, and transportation sectors [2]. In industrial automation, TSN is crucial for the real-time control of industrial robots and other automation equipment, ensuring the efficient operation of production lines. To support intelligent manufacturing, TSN facilitates real-time communications between production equipment, providing essential support for advanced manufacturing processes. Additionally, TSN is widely employed in autonomous vehicles, trains, and aircraft to ensure effective cooperation and communication among systems, thereby enhancing both traffic safety and efficiency [3]. Here, it can also be integrated with recognition and control algorithms [4,5,6] to improve the transmission speed in a traffic signal system, thereby enabling more efficient transportation management. Therefore, TSN plays a pivotal role in shaping the future of industrial networks, providing the reliable communication necessary for the advancement of IIoT applications.

TSN is an Ethernet technology standard aimed to meet the real-time, predictable, and low-delay requirements in various domains [7]. Its standard comprises a series of specifications under the IEEE 802.1 [8] umbrella, which can be categorized into four main areas: time synchronization, scheduling, reliability, and resource management. These encompass a range of protocols including IEEE 802.1AS, IEEE 802.1Qbv, and IEEE 802.1Qbu, among others. Built upon the traditional Ethernet, these protocols establish novel networks with time-sensitive characteristics, enabling time synchronization, bounded ultra-low-delay reliable transmission, and deterministic communication [7]. TSN clearly defines three distinct traffic types: Best-effort (BE), Audio–Video Bridging (AVB), and Time-triggered (TT) traffic. Among these, TT traffic imposes strict delay requirements and thus holds the highest priority. To ensure the predictable end-to-end transmission delay for TT traffic, TSN employs traffic shaping and scheduling mechanisms such as the Time-Aware Shaper (TAS) and Cycling Queuing and Forwarding (CQF). Initially, TSN synchronizes the local clocks of all bridges and end stations in the network [9] to ensure global time synchronization [10]. Subsequently, Centralized Network Configuration (CNC) schedules TT traffic across different bridges based on static traffic scheduling algorithms [7]. This scheduling process is pre-computed to guarantee precise temporal control over data transmission within the network. These mechanisms effectively meet the communication requirements of time-critical applications, maintaining bounded low-delay transmission within a controllable range.

Both TAS and CQF methods aim to shape traffic at ingress and egress by performing enqueue and dequeue operations under global time synchronization to ensure the deterministic delivery of end-to-end flows. In the TAS [11], two switch signals (0 and 1) are utilized to control each queue connected to the relevant egress port. These control signals are generated based on a predefined scheduling scheme called a Gate Control List (GCL), which requires dynamic configuration for each queue of all network hosts and switches. The TAS achieves microsecond-level fine-grained scheduling on a per-hop, per-packet basis. However, its operation necessitates the configuration of the GCL, leading to significant complexity. To address the issues associated with the TAS, TSN proposed CQF as an alternative traffic-shaping method. In CQF [12], packets are classified based on their arrival time and sequentially transmitted to the respective odd and even queues without considering the input and output times of packets. By smoothly controlling packet arrival times and rates, traffic shaping and periodic forwarding are achieved, ensuring the delay jitter performance of critical flows. This method effectively accomplishes traffic shaping and partially resolves the challenges of complex GCL generation and QoS analysis. However, there still exist some challenges in CQF. Firstly, determining an appropriate cycle time is an important issue, as long cycles lead to an increased delay while short cycles increase bandwidth requirements [13]. Secondly, CQF employs two buffers, one for receiving and one for transmitting, which cannot be simultaneously filled and emptied [14]. To overcome these challenges, researchers have proposed extensions to CQF. One such extension is Cycle-Specified Queueing and Forwarding (CSQF) [15], which considers three buffers instead of two. CSQF relaxes the constraint of the same cycle arriving at the next switch, easing link delay constraints. However, it also necessitates planning cycle offsets for traffic at each node to ensure compliance with the storage resource constraints of switches, exponentially increasing the complexity. Additionally, there is a combination method consisting of second-layer local CQF and third-layer wide-area CSQF [16]. Recently, Finn proposed Multi-CQF as an extension of CQF and discussed the pros and cons of various CQF variants [13]. However, in the original Multi-CQF, the scheduling algorithm lacks flow prioritization assignment and performs weakly in handling dynamic changes during traffic transmission.

In the context of the IIoT, real-time and deterministic communication over Multi-CQF networks is essential. Efficient traffic scheduling plays a critical role in ensuring timely data transmission across industrial devices. This work is motivated by the need to develop scalable and effective scheduling strategies that can meet the stringent requirements of IIoT applications while maintaining computational efficiency. To construct an effective traffic scheduling model based on Multi-CQF, this paper first establishes the traffic scheduling model of Multi-CQF according to queuing theory. In addition, two optimization strategies are proposed to address the scheduling optimization problem of Multi-CQF for different traffic networks. Mixed Integer Programming (MIP) continuously searches the feasible solution space through branch-and-bound and linear programming techniques, updating the optimal solution and upper bound during the search process. However, it is only suitable for small-traffic TSN networks due to its limited scalability. For large-traffic TSN networks, the Variable Neighborhood Search Genetic Algorithm (VNS-GA) utilizes genetic operations to preserve and transmit excellent genetic information, introduces new variations to promote diversity and exploration, and alters the local structure of the solution through a variable neighborhood search. This combination effectively balances global and local searching, facilitating the discovery of high-quality scheduling solutions. Compared with other existing schemes, computer simulation reveals that in small-traffic TSN networks, MIP reduces the average delay time by approximately 13%, while in large-traffic TSN networks, the VNS-GA can reduce the average delay time by about 7%.

The main contributions of this paper are summarized as follows:

1. For Multi-CQF, a novel traffic queuing model has been devised to enhance efficient flow transmission and resource utilization. This proposed model helps maintain load balancing throughout the scheduling process, thereby improving overall resource utilization on a global scale.
2. A scheduling generation tool based on an MIP solver has been developed. This proposed method leverages the precision and efficiency of MIP to explore optimal scheduling schemes within search processes of small-traffic networks.
3. In large-traffic networks, the use of the VNS-GA for addressing combinatorial optimization problems aims to discover superior scheduling schemes. By iteratively altering search neighborhoods, this proposed method mitigates the risk of converging to local optima and enhances its efficiency and performance.

The remainder of this paper is organized as follows. Section 2 presents an overview of the Multi-CQF. In Section 3, the proposed scheduling method is provided, along with the MIP and VNS-GA-based optimization strategies of Multi-CQF. Section 4 presents the simulation results and analysis. Finally, Section 5 concludes the paper and outlines the future research direction.

## 2. Related Work

In recent years, researchers have proposed various scheduling models for TSN. Crăciună et al. [17] provided an in-depth explanation of the IEEE 802.1Qbv standard and conducted detailed research, presenting a Satisfiability Modulo Theory (SMT) model based on linear integer algorithms. This model identifies precise transmission offsets for each frame to minimize the number of queue utilizations. They further observed that a Generalized Credit-Based Shaper (CBS) without length restrictions is impractical for real-world devices. Consequently, they introduced a set of window-based GCL scheduling constraints, mapping frames to windows [18]. Oliver et al. [19] reformulated these constraints [18] into an SMT model based on array theory, with the objective of minimizing jitter. They experimentally evaluated and compared this window-based synthesis algorithm with the frame-based [17] one using the Z3 v4.4.1 solver (64bit). Additionally, they employed integer linear programming (ILP) to solve scheduling tasks [20]. Nayak’s research [21] modeled the scheduling problem as a no-wait packet scheduling problem and employed a proposed tabu search-based heuristic algorithm, previously applied to no-wait job shop scheduling problems [22]. The objective was to allocate as much contiguous space as possible for BE traffic.

**Figure 1:**
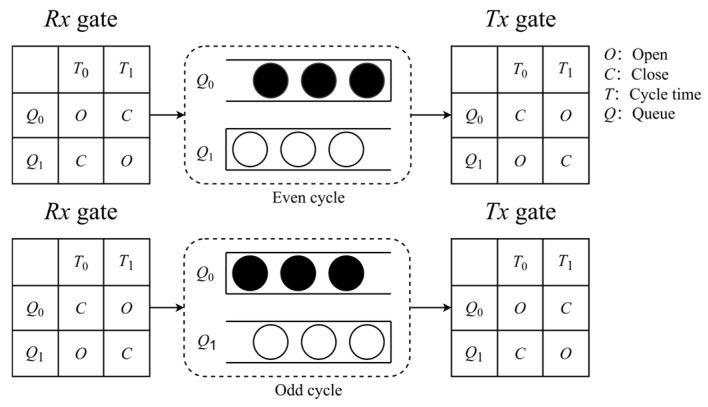
CQF queue scheduling mechanism in even and odd cycles.

CQF employs a ping-pong queue model [23] to schedule traffic, ensuring deterministic delay communication. As depicted in Figure 1, the original CQF model utilizes two queues, denoted as *Q*_0_ and *Q*_1_. Figure 1 assumes two time intervals, *T*_0_ and *T*_1_, within one time cycle. The receiving gate (*Rx*) and transmitting gate (*Tx*) control the queue reception or transmission of flows. When the *Rx* or *Tx* gate is open (*O*), the associated queue can receive or transmit flows. Conversely, when the *Rx* or *Tx* gate is closed (*C*), the associated queue prohibits the reception or transmission of flows. For instance, during the odd time slot within interval *T*_0_, *Q*_0_ opens its *Rx* gate to receive traffic, while *Q*_1_ opens its *Tx* gate to transmit traffic. Conversely, during the even interval *T*_1_, the operations are reversed, cyclically alternating the transmission states of the two queues.

In the research of CQF scheduling, there exist two scheduling approaches, offline and online scheduling. For offline scheduling, J. Yan et al. [24] addressed the traffic scheduling problem and proposed the ITP mechanism to optimize the network throughput of time-sensitive flows. ITP effectively maps time-sensitive flows to CQF queues by adjusting the time slots for each flow. Furthermore, they designed a tabu-inspired heuristic algorithm with domain-specific optimization strategies for traffic scheduling. To enhance the scheduling performance of CQF, Y. Zhang et al. established the FLJ-VB algorithm based on the divisibility theory [25]. This algorithm ensures load balancing between periodic and non-periodic flow transmissions while further reducing scheduling complexity. Regarding online scheduling for CQF, W. Quan introduced the Flow Injection Time Scheduling (FITS) algorithm [26] by generating traffic scheduling increments for new time-sensitive flows. As standalone CQF cannot support mixed traffic transmission, Finn [13] proposed Multi-CQF as an extension of CQF. Konstantinos [27] optimized the traffic scheduling of Multi-CQF by utilizing Constraint programming (CP) formulations and Simulated Annealing (SA)-based metaheuristic solutions, demonstrating its effectiveness even under tight timing constraints. However, with the increase in network traffic, both CQF and Multi-CQF may lead to greater time delays. This paper primarily tackles offline scheduling problems by integrating queueing theory with heuristic algorithms to optimize the performance of Multi-CQF based traffic scheduling systems.

## 3. The Proposed Scheme

In this section, we present a comprehensive overview of the proposed TSN scheduling architecture, elucidating the system model, problem formalization, and scheduling methods and optimization strategies in detail.

### 3.1. System Model

#### 3.1.1. Architecture Model

**Figure 2:**
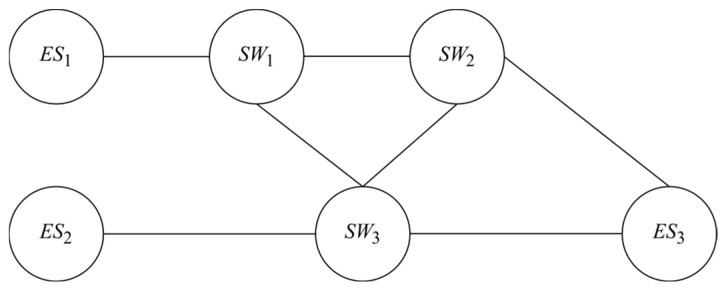
Example topology of a TSN-based network.

The network topology depicted comprises three end devices (*ES_i_*) and three TSN switches (*SW_i_*). As illustrated in Figure 2, the TSN switches (*SW_i_*) are interconnected via physical links. An *ES_i_* can serve as either the source or the destination of traffic, while *SW_i_* facilitates data forwarding. The network architecture is assumed as a directed graph *G* = {*V*, *E*}, where *V* represents the set of all nodes, and *E* represents the set of transmission links on the network, as shown in Figure 2.

#### 3.1.2. Traffic Model

In TSN, traffic types include the following: (1) TT flow is primarily utilized within the IIoT domain, intended for conveying industrial control flows with stringent real-time and deterministic requirements. It is assigned the priority level as *P_TT_*. (2) AVB flow is primarily designated for bandwidth-intensive audiovisual flows and certain delay-sensitive services. Its priority is lower than TT’s, denoted as *P_AVB_*. (3) BE flow is mainly applied to services with relaxed delay requirements, occupying the lowest priority level, designated as *P_BE_*. The priority sequence for these traffic types is *P_TT_* > *P_AVB_* > *P_BE_*.

#### 3.1.3. Multi-CQF

**Figure 3:**
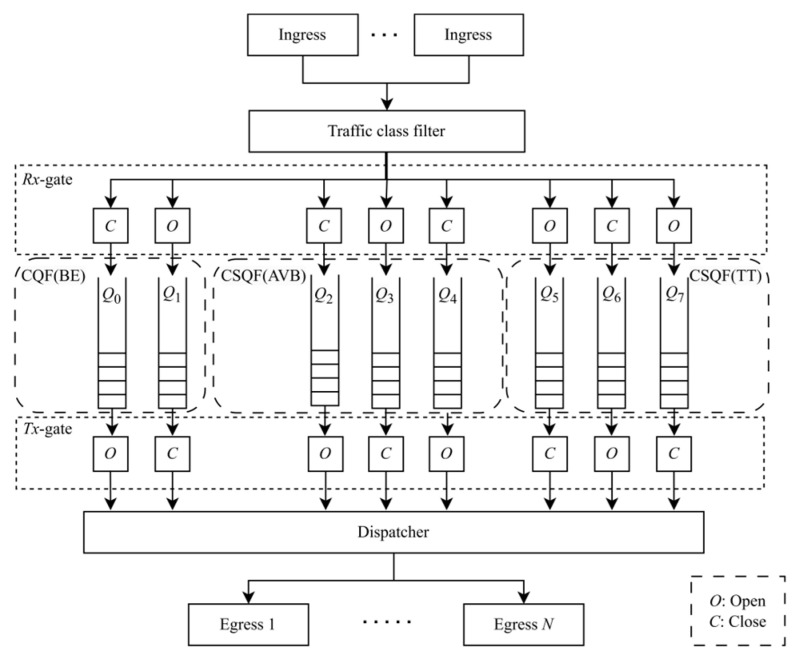
An example of a TSN switch using Multi-CQF.

As the initial shaper, CQF employs only two queues. All variants of CQF operate on a fixed-length cyclic basis. During odd cycles, one queue buffers frames received at the input ports, while the other queue transmits frames cached by the previous queue of the even cycle. In even cycles, the roles of the queues are swapped. Figure 3 illustrates an example of a Multi-CQF switch architecture, where a CQF shaper and two CSQF shapers together constitute a Multi-CQF. Here, BE traffic utilizes the CQF shaper, while AVB and TT traffic utilize the CSQF shapers.

Considering the example depicted in Figure 3, we define the superperiod, *H*, as the length of a cyclic time period, typically referring to the least common multiple (LCM) of the flow periods. We have a total of eight queues. Queue 0–1, 2–4, and 5–7 are utilized for the transmission of BE, AVB, and TT traffic, respectively. Furthermore, there exist three priority groups, denoted as *P* = {*P_BE_*, *P_AVB_*, *P_TT_*}. *P_BE_* comprises two queues (*MQ_BE_* = {*Q*_0_, *Q*_1_}) with a cycle length ||*C*_1_|| = 125 μs. *P_AVB_* consists of three queues (*MQ_AVB_* = {*Q*_2_, *Q*_3_, *Q*_4_}) with a cycle length ||*C*_2_|| = 250 μs. *P_TT_* encompasses three queues (*MQ_TT_* = {*Q*_5_, *Q*_6_, *Q*_7_}) with a cycle length ||*C*_3_|| = 250 μs. The cyclic period *H* = 250 μs.

### 3.2. Problem Formulation

In this paper, we investigate the problem of minimizing end-to-end delay. Without proper planning of flow transmission within switch queues, it can lead to traffic loss due to queue congestion and network overload, resulting in increased delay. We formalize the scheduling issue as an optimization problem, incorporating four constraints and a single objective function.

#### 3.2.1. Objective Function

The objective function is to minimize the average end-to-end delay of all flows, where the end-to-end delay is the sum of queuing delay and transmission delay in the time domain. The average end-to-end delay is defined by Equation (1). [*v_a_*, *v_b_*] represents the path of flow *i* from endpoint *a* to endpoint *b*, where *i* ∈ *I*. *Delay*(*q*) denotes the queuing delay of flow *f_i_*, and *Delay*(*t*) denotes the transmission delay of flow *f_i_*.

(1) $$minE2E(i)=\frac{1}{\left|I\right|}\sum_{i=0}^{I}\left(\;f_{i}^{\;\left[v_{a},v_{b}\right]}\_\mathrm{Delay}(q)+f_{i}^{\;\left[v_{a},v_{b}\right]}\_\mathrm{Delay}(t)\right)$$

#### 3.2.2. Offset Constraint

The offset constraint stipulates that the initiation time of a data flow should fall within its time slot. Otherwise, multiple frames of the same flow would accumulate within the switch, consuming the limited buffer size. For example, the packets in the first period of a flow have been forwarded before that generated in the second period. For a set of flows *F =* {*f*_0_,…, *f_n_*_−1_}, the start time of any flow *f_i_* ∈ *F* must be controlled within a reasonable time range to avoid data frame backlog and resource waste. Given that the granularity of the offset is *T_slot_*, *f_i_*(*period*) must be divided by the length of one time slot. The offset constraints are formulated in Equations (2) and (3).

(2) $$\forall f_{i}\in F,\;i\in \left[0,n-1\right]$$

(3) $$0\;{\leq f}_{i}(\mathrm{offset})<\frac{f_{i}(\mathrm{period})}{T_{\mathrm{slot}}}$$

#### 3.2.3. Term Constraints

In all data flows, each flow is associated with a deadline. If the start time of a particular flow is delayed, it cannot reach its destination on time. To mitigate this scenario, this constraint dictates that all flows must arrive before their respective deadlines. The term constraint is formulated in Equation (4). Here, *hop_num_*(*f_i_*) represents the number of switches along the flow path.

(4) $$f_{i}(\mathrm{offset})+{\mathrm{hop}}_{\mathrm{num}}(\;f_{i}\;)\leq \frac{f_{i}(\mathrm{deadline})}{T_{\mathrm{slot}}}$$

#### 3.2.4. Time Slot Constraints

The slot constraint restricts the maximum and minimum length of a slot. Given that the unit of offset is the slot in Multi-CQF, it is imperative to ensure that the periods of all flows are divisible by the length of the slot. Thus, the maximum slot length corresponds to the greatest common divisor (GCD) of all flow periods, as illustrated by Equation (5):

(5) $$\mathrm{Max}(T_{\mathrm{slot}})=\mathrm{GCD}[\;f_{i}(\mathrm{periods})]$$

In practical applications, *Min*(*T_slot_*) is significantly smaller than *Max*(*T_slot_*). *T_slot_* is a property of the network in TSN based on Multi-CQF, which needs to be predetermined as a constant.

### 3.3. Scheduling Methods and Optimization Strategies

In this section, the traffic scheduling solution is proposed along with the optimization strategies employed.

#### 3.3.1. Traffic Scheduling Model Based on Queuing Theory

For the traffic scheduling problem, we initially employ queueing theory to select appropriate queues and determine the characteristics of frames for each flow. Based on these characteristics, the optimal scheduling order for frames can be achieved through optimization formulas or heuristic algorithms to meet specific performance metrics or objectives. We conduct an in-depth analysis of network topology, traffic characteristics, and resource allocation to identify the optimal traffic scheduling strategy. Optimization of resource utilization is attained through performance metrics of queueing systems.

**Figure 4:**
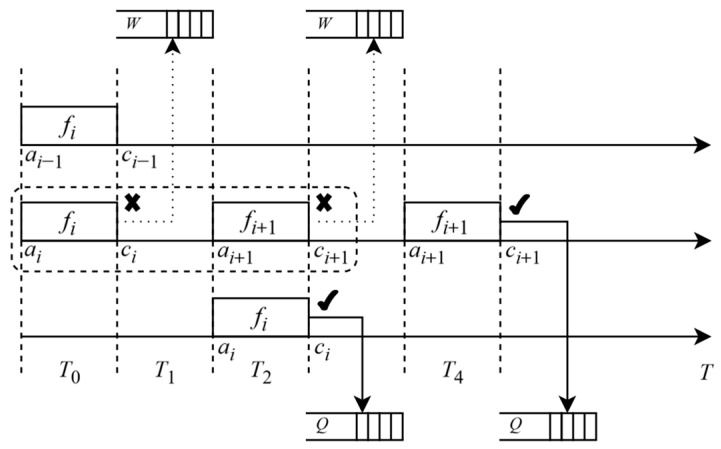
Queuing theory-based traffic scheduling.

For each frame *i*, assume that *a_i_* denotes the arrival time of the *i*th frame, *c_i_* represents the completion time of the *i*th frame, *Q* denotes the queue, and *W* represents the waiting queue. For *i*, if *a_i_* > *c_i_*_−1_, it indicates that the current *i* can immediately begin execution since its arrival time exceeds the completion time of the previous frame. At this point, we assign *i* to an available *Q* for transmission, as illustrated by the solid box between *f_i_* and *f_i_*_+1_, or if all queues are busy, it is placed in *W*, as depicted by the dashed box between *f_i_* and *f_i_*_+1_. If *a_i_* < *c_i_*_−1_, it indicates that the current *i* needs to wait for the previous frame task to be completed. Subsequently, *i* is added to *W*, where frames in the waiting queue will resume processing after the completion of the preceding frame task, as illustrated in Figure 4.

#### 3.3.2. MIP-Based Traffic Scheduling in Small-Traffic TSN

MIP extends linear programming (LP) and is commonly employed to find an optimal solution within given constraints. In this study, MIP allows the existence of both integer and continuous variables, so scheduling problems that include both discrete and continuous decisions can be expressed flexibly. MIP is utilized to tackle the traffic scheduling problem in small-traffic TSN networks. The primary advantage of MIP over LP lies in its ability to handle integer decision variables, making it well-suited for addressing practical and complex optimization problems that involve discrete decisions. Moreover, MIP accommodates both integer and continuous variables within a single optimization model, offering greater precision and flexibility in problem-solving. The solving process can be delineated into the following steps.

*Step* 1 (Branching): Let *L* = {*P*(*X*)}, where *P*(*X*) represents the sub-problem of decomposing the network flow scheduling problem into the selection of paths. *L* denotes the set of all sub-problems. Each sub-problem selects a set of paths for transmitting traffic and optimizes the delay time along the paths. We obtain a feasible point x* as initially selected paths generated from queuing theory-based solutions, and v* = *f*(x*) which represents the delay time under x*. If there is no initial feasible solution, v* = +∞.

*Step* 2: If *L* ≠ Ø, select one or more nodes from *L*, denoted as *L* = {*P*(*X*_1_),…,*P*(*X_k_*)}, and proceed to *Step* 3 with *i* = 1 (*i* < *k*), i.e., select one sub-problem to solve. Otherwise, if no sub-problems remain, terminate the procedure and then go to *Step* 5.

*Step* 3 (Bounding and update): Calculate the lower bound *LB_i_* of sub-problem *P*(*X_i_*). If *LB_i_* < v*, proceed to *Step* 4. If the optimal solution of *P*(*X_i_*) yields a shorter delay time than the current best feasible solution v*, search for feasible solutions, updating x* and the upper bound v* if found.

*Step* 4 (Pruning): Remove *P*(*X*_i_) from *L*. If *i* < *k*, increment *i* by 1 and return to *Step* 3; otherwise, proceed to *Step* 2.

*Step* 5: Output the optimal scheduling solution, *S* = v*.

**Figure 5:**
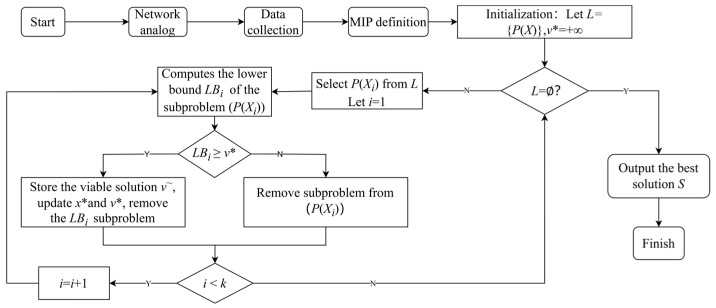
Flow chart of MIP algorithm.

The feasible region of variables is partitioned into two or more sub-problems, dividing the search space into disjoint subspaces. Subsequently, each sub-problem is addressed individually. Following the branching, the solver handles the segregated sub-problems, each of which constitutes an independent optimization problem. These sub-problems are a linear programming problem, employing the objective function and constraints of the original problem along with additional branching constraints such as variable bounds. The solver endeavors to solve each sub-problem to identify the optimal solution or enhance the lower bound (*LB*), progressively narrowing down the search space. The next step involves determining the upper bound (*UB*) and the lower bound (*LB*), where the *UB* is an estimate of the known optimal solution, usually initialized as positive infinity. The *LB* represents the current best estimate of the optimal solution. Our objective is to gradually diminish the *LB* to approach the *UB* (branch-and-bound). When the *LB* sufficiently approximates the *UB*, the algorithm can terminate, indicating that the optimal solution has been found. Pruning is a pivotal component of the branch-and-bound algorithm. If during the sub-problem solving it is observed that the *LB* exceeds the current *UB*, the sub-problem can be pruned, ceasing further exploration. This is because a superior solution is deemed unattainable in this sub-problem, thus it can be safely disregarded, reducing the search space. Pruning operations can significantly enhance the efficiency of the algorithm. The specific procedural diagram is depicted in Figure 5.

For small-traffic problems, MIP methods typically produce precise optimal solutions. However, as network traffic increases, the problem complexity often results in excessively long solution times and a greater risk of convergence to local optima. In contrast, heuristic algorithms are better suited for large-traffic problems, as they provide high-quality, feasible solutions within reasonable time frames. Therefore, heuristic algorithms are necessary for certain problems where obtaining exact optimal solutions would require prohibitive computational resources.

#### 3.3.3. VNS-GA-Based Traffic Scheduling in Large-Traffic TSN

Several metaheuristic algorithms have been proposed in the literature [25]. The aim of these algorithms is to find a high-quality solution within a reasonable time frame, without guaranteeing optimality. Building upon the review of relevant works, we introduce the VNS-GA designed to effectively reduce end-to-end delay in traffic transmission. The GA is well-suited for discrete search spaces, whereas most other metaheuristic algorithms are primarily designed for continuous domains. Since the problem addressed in this paper is inherently discrete, the GA is adopted as the core optimization approach. Furthermore, the integration of a VNS strategy into the GA significantly enhances its local search capability and convergence performance.

**Figure 6:**
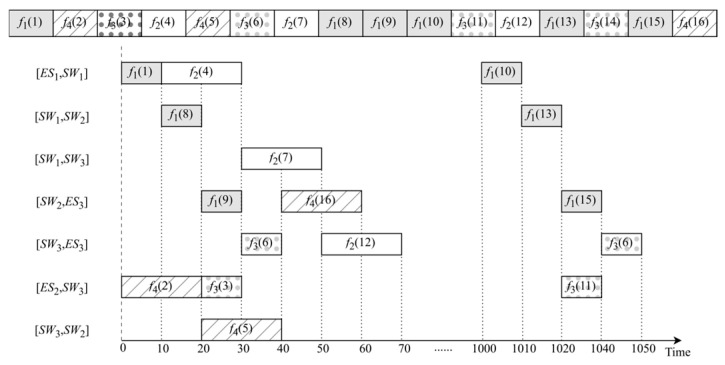
Time diagram of chromosome generation.

In the context of the GA, the solution to scheduling optimization problems is represented as chromosomes composed of genes. The optimal chromosome for problem-solving is identified through operations such as evolution and mutation. Chromosomes comprise frames represented as genes, with each frame initially generated using queuing theory. In Gantt charts, frames are placed on source-to-destination paths according to the sequence of frames on the chromosome, thereby generating a timeline. When a frame is scheduled on a particular path, scheduling on the next path should commence from the completion time of transmission on the preceding path. By iteratively following this approach, the timelines for all genes in the chromosome can be generated. As shown in Figure 6, a chromosome is composed of four different types of traffic spliced into frames. For example, since *f*_1_(1) is the initial frame in Figure 6 (the numbers in parentheses represent the serial number in the chromosome, and the subscripts represent which traffic its frame belongs to), it is positioned on [*ES*_1_, *SW*_1_] following the route specified in Table 1. As an example of frame properties, *f*_4_(2), indicated as the second gene, is placed on [*ES*_2_, *SW*_3_]. Similarly, *f*_3_(3) as the third gene is placed on the same link. Table 1 provides an example of transmission cycles, transmission delays, and routing in the network.

Firstly, in the genetic phase, the population represents various scheduling schemes. The population consists of chromosomes, with each chromosome representing a particular scheduling solution.

*Step* 1: Generate an initial population, as shown in Equation (6).

(6) $${\mathrm{pop}}_{i}(t),\;i=1,2,3,\ldots ,n$$

*Step* 2: Calculate the fitness values.

Assume that the fitness function is denoted as *f*(*i*). Calculate the corresponding values for all individuals, as shown in Equation (7).

(7) $$f(i)=f({\mathrm{pop}}_{i}(t))$$

*Step* 3: Select chromosomes for crossover operation.

Chromosomes with higher fitness values are chosen for crossover operation. The selection probability for each chromosome is denoted as *P_i_*, as shown in Equation (8).

(8) $$P_{i}=\frac{f(i)}{\sum_{i=1}^{n}f(i)},\;i=1,2,3,\ldots ,n$$

Recombine *P_i_* as the selection probability of the *i*th chromosome and *C_i_* as the *i*th selected chromosome into a new population *popnew* (*t* + 1), as shown in Equation (9).

(9) $${\mathrm{pop}}_{\mathrm{new}}(t+1)=\sum_{i=1}^{{\mathrm{pop}}_{i}(t)}P_{i}\cdot C_{i}$$

*Step* 4: Execute crossover operation.

In the crossover operation, we determine whether an individual is chosen for crossover based on their relative fitness values. A tournament selection method is employed, randomly selecting two individuals and comparing their fitness values. The superior individual is then selected for the crossover operation, resulting in a new population, cross *pop* (*t* + 1). Here, Partially Mapped Crossover (PMX) is introduced as the crossover method.

**Figure 7:**
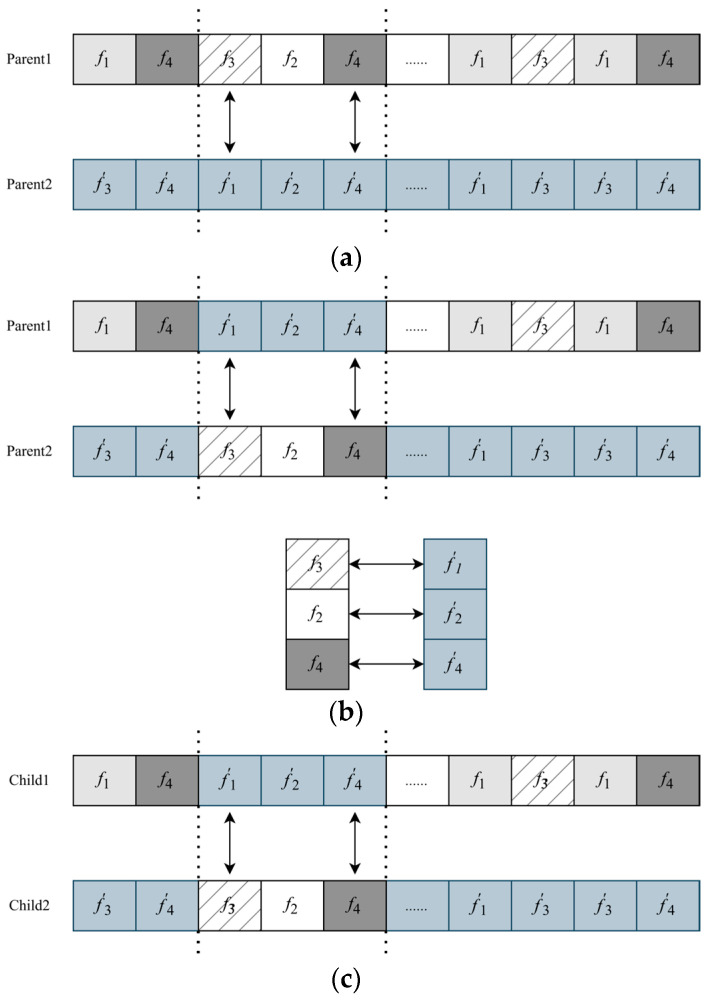
PMX operation. (**a**) Selection of gene fragments with identical start and end positions. (**b**) Exchange of gene fragments. (**c**) Conflict detection and mapping between exchanged gene fragments.

Figure 7 illustrates an instance of the PMX operation. This operation preserves the positions of a specific number of genes in the chromosome. A pair of chromosomes, *f_n_* and *f*′*_n_* (parents), are randomly selected from the current generation, and three gene segments with identical start and end positions are chosen, as depicted in Figure 7a. These two segments are then swapped, as shown in Figure 7b. Subsequently, conflict detection is performed to establish a mapping between the exchanged gene segments, as illustrated in Figure 7c. Through this mapping, the formation of new pairs of offspring genes is ensured to be conflict-free. PMX guarantees that the generated offspring chromosomes do not exhibit duplicate or missing genes, thereby facilitating the rational selection of traffic paths. By employing partial matching, the offspring chromosomes encompass complete genetic information from both parents, ensuring the diversity and integrity of traffic path selection in the TSN network.

*Step* 5: Execute mutation operation.

Randomly select individuals from the population and perform chromosome mutation with a mutation probability *P_m_* = 0.05 to obtain a new population, *mutpop* (*t* + 1), as shown in Equation (10). This population serves as a subpopulation for completing one genetic operation, i.e., *pop*(*t*) = *mutpop* (*t* + 1). Proceed to *Step* 2 at this point.

(10) $$\mathrm{mutpop}\;(t+1)=\{{\;\mathrm{pop}}_{j}(t)|j=1,2,3,\ldots ,n\}$$

*Step* 6: Define neighborhood structures.

Firstly, define a neighborhood structure *N__k_*(*x_m_*) for the Shaking Procedure, which utilizes random perturbations to alter transmission paths, as shown in Equation (11). Secondly, introduce a neighborhood structure *N__l_*(*x_n_*) for the Variable Neighborhood Descent (VND) process, enabling exploration across multiple distinct search spaces, thereby enhancing the likelihood of finding the global optimum, as shown in Equation (12). Select the top 30% of fittest individuals from *pop*(*t*) to serve as initial solutions for the variable neighborhood operations.

(11) $$N_{\_k}(x_{m})=\{x_{m}|x_{m}=x_{m-1}+\varphi ,\;\varphi \in R^{n},\;m=1,2,3,\ldots ,k\}$$

(12) $$N_{\_l}(x_{n})=\{x_{n}|x_{n}={\mathrm{neighbor}}_{n-1}(x_{n-1}),\;n=1,2,3,\ldots ,l\}$$

In *N__k_*(*x_m_*), *x_m_* is the solution after perturbation, φ is a disturbance factor. For *N__l_*(*x_n_*), *x_n_* represents a new solution generated by *x_n_*_−1_ from neighborhood operations (exchange, 2-Opt, and or-Opt) of *Step* 7.

*Step* 7: Execute neighborhood operations

**Figure 8:**
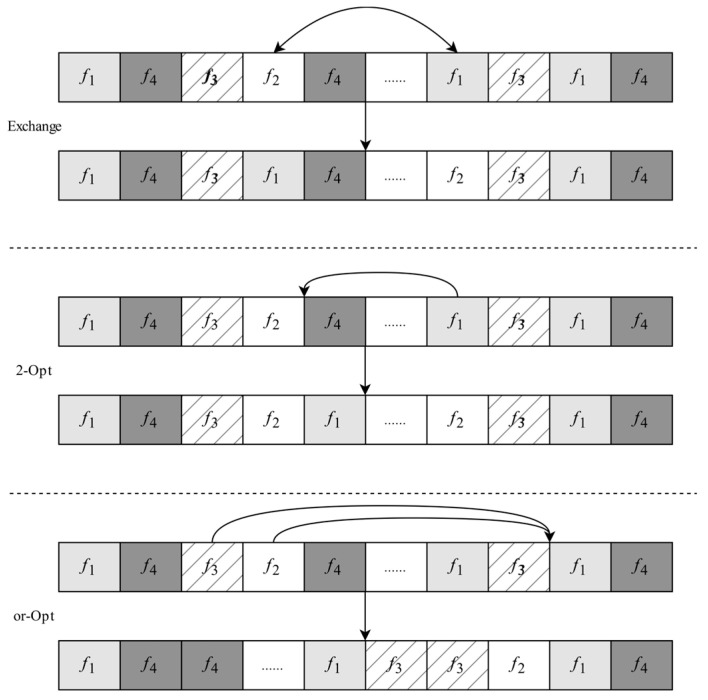
Three types of neighborhood operators.

As shown in Figure 8, three types of operators designed for variable neighborhood search are exchanged, 2-Opt and or-Opt, respectively. Here, three operators are applied sequentially. The exchange operator randomly selects two different frames from the current solution and exchanges their positions. The 2-Opt operator randomly selects two different frames, such as *f*_1_ and *f*_2_, and reverses the order of the nodes after frame *f*_2_. The or-Opt operator randomly selects two consecutive nodes, such as *f*_2_ and *f*_3_, and reverses their order, inserting them after a randomly selected node, *f*_3_. Set the initial domain structure, select the first domain operation (exchange operator), and search locally in the current domain structure. If the solution found in the current neighborhood is better than the current solution, then accept the solution, and go to the next neighborhood operations (2-Opt the and or-Opt operator) until no better solution is found in all the neighborhood structures or the predefined condition is satisfied.

*Step* 8: Reinsert the set of local optimal solutions back into the original population and proceed to *Step* 2.

*Step* 9: Output results.

Compare the current iteration count (*gen*) with the maximum iteration count (*Max gen*). After each iteration, the algorithm compares *gen* with *Max gen*. If *gen* < *Max gen*, the algorithm proceeds to *Step* 4; otherwise, the algorithm terminates and outputs the solution *S* with the highest fitness.

**Figure 9:**
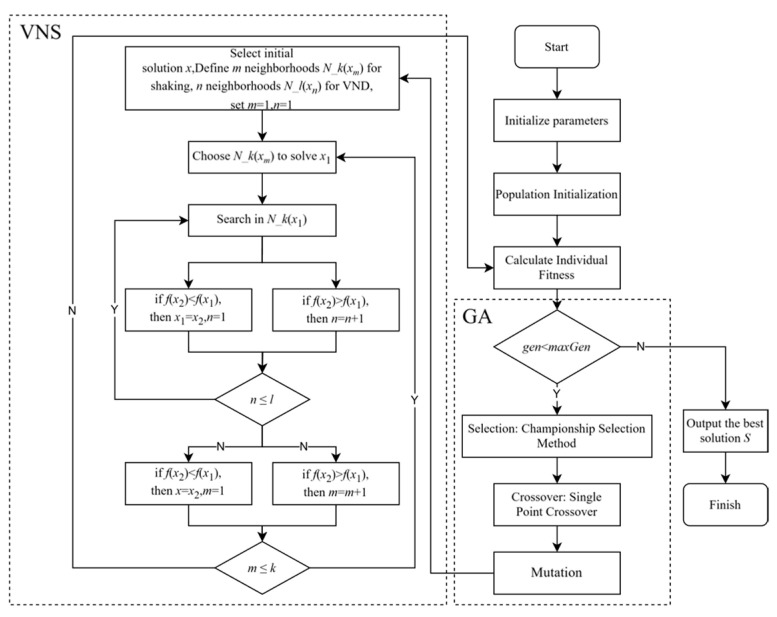
Flowchart of the VNS-G.

The genetic component in the VNS-GA utilizes population evolution to globally explore the solution space, while the variable neighborhood search continually adjusts the neighborhood of solutions for local exploration, aiming to identify superior solutions. By synergistically employing these two strategies, the VNS-GA achieves an optimal balance between global and local search, facilitating the rapid discovery of high-quality solutions. The specific process is illustrated in Figure 9.

## 4. Performance Evaluation

Computer simulation is conducted to demonstrate the effectiveness of the proposed algorithms. Additionally, the statistical analysis of the results was performed by referring to the method presented in [28]. The MIP mathematical optimization model is formulated in python, using COIN-OR as the linear programming module. Similarly, the VNS-GA-based Multi-CQF is also implemented in Python 3.8.0. All simulations are performed on a PC equipped with an NVIDIA GeForce RTX 3060 Laptop GPU(Lenovo Group Limited, headquartered in Beijing, China). The parameter settings of the VNS-GA are summarized in Table 2.

### 4.1. Simulation Environments

We referenced the typical characteristics of industrial automation network standards [29] (IEC/IEEE 60802) to describe the features of flows. All processes in our simulations are randomly generated under the guidance of the IEC/IEEE 60802 standard to facilitate better comparison, with varying numbers of flows generated. We set the link rate to 1 Gbps and the global time slot size to 125 μs. Considering the queue lengths of different priority levels, the queue lengths of *P_TT_*, *P_AVB_*, and *P_BE_* are set to 8 K, 4 K, and 4 K, respectively. The source and destination of each flow are randomly selected from *ES*s. For TT traffic-type flows, the flow period is selected from {1000, 2000, 5000, 10,000} μs, and message sizes are randomly generated between 50 and 500 B. For the *AVB* and *BE* traffic types, the flow period is selected from {100, 500, 1000, 1500, 2000} μs, with message sizes randomly generated between 30 and 100 B.

### 4.2. Simulation Results

The delay, resource utilization rate, resource distribution variance, and execution time are crucial metrics for evaluating algorithm performance. Delay reflects the responsiveness of the system. The resource utilization rate measures the efficiency of system usage. The resource distribution variance evaluates the load balance across different resources. Execution time represents the computational efficiency of the algorithm. This section will compare and analyze the performance of the proposed algorithms (MIP and the VNS-GA) against existing algorithms (i.e., Constraint programming (CP) [13], Simulated Annealing (SA) [13], Naive greedy (NG) [19], and Naive (NV) [20]) in these four aspects. The main characteristics of the four existing algorithms are summarized in Table 3.

#### 4.2.1. Latency with Different Iterations in the VNS-GA

**Figure 10:**
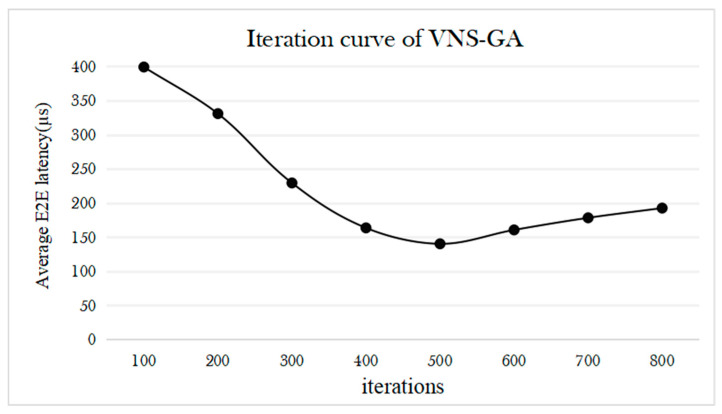
Iteration curve of the VNS-GA.

Figure 10 illustrates the iteration curve of the proposed VNS-GA. Simulation results indicate a significant reduction in average delay as the iteration count reaches 500, suggesting effective convergence to a relatively optimal solution in the early stage. However, as the iteration count continues to increase, the optimization effect gradually diminishes, possibly due to the algorithm becoming trapped in local optima. This trend becomes more evident as the search space narrows and convergence decelerates. Therefore, all subsequent simulations are conducted under the condition of 500 iterations to balance iteration count and optimization effectiveness, aiming to achieve the optimal optimization outcome.

#### 4.2.2. Average Delay in Small-Traffic TSN Network

**Figure 11:**
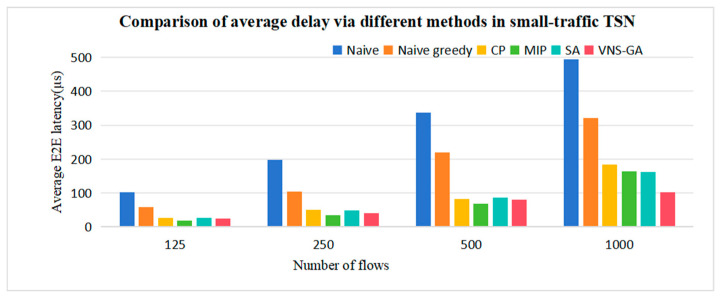
Comparison of average delay via different methods in small-traffic TSN.

We conducted comparisons of the performances of CP and MIP in Multi-CQF across different traffic TSN networks, as well as the capability of heuristic algorithms to find high-quality solutions. As depicted in Figure 11, the results indicate that the proposed MIP algorithm reduces an average delay by approximately 76% and 58% compared to Naive and Naive greedy, respectively. In contrast to the CP formula, the MIP algorithm exhibits significant scheduling advantages in small-traffic networks, enabling the identification of global optimal solutions, resulting in an average latency reduction of approximately 13.33%. However, as the TSN network scales up, with the number of flows exceeding one thousand, the MIP algorithm is outperformed by the SA and VNS-GA. This is due to the escalating complexity of the problem in large-traffic networks, which may result in exceedingly long solution times for MIP algorithms and a higher likelihood of becoming trapped in local optima. Conversely, heuristic algorithms boast lower computational complexity, enabling faster approximation to optimal solutions.

#### 4.2.3. Average Delay in Large-Traffic TSN Network

**Figure 12:**
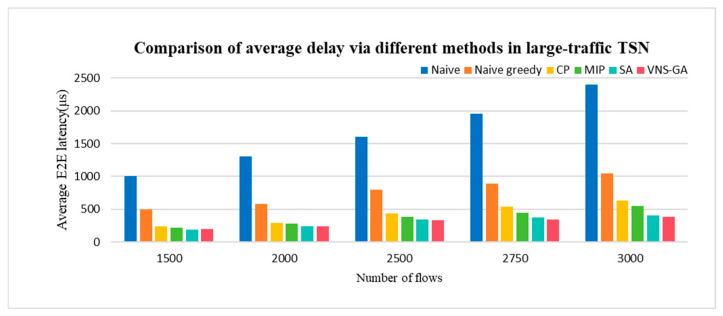
Comparison of average delay via different methods in large-traffic TSN.

As anticipated, heuristic algorithms consistently achieve superior delay performance with the increase in flow volume. Large-traffic networks typically comprise numerous nodes and edges, rendering exact methods such as MIP computationally prohibitive. In such scenarios, heuristic algorithms often present a more viable alternative. In comparison to Naive, Naive greedy, CP, and MIP approaches, both SA and the VNS-GA yield commendable optimization outcomes. With combining diverse search strategies, the proposed VNS-GA method facilitates a more comprehensive exploration of the solution space, thus converging to near-optimal solutions more expediently. Consequently, the proposed method outperforms SA by approximately 7.44% and demonstrates notable robustness in large-traffic TSN networks. Simulation findings are depicted in Figure 12.

#### 4.2.4. Resource Utilization

The resource utilization rate (*U_i_*) refers to the proportion of the allocated queue (*Q_i_*) to the total queue (*Q_total_*), as shown in Equation (13).

(13) $$U_{i}=\frac{Q_{i}}{Q_{\mathrm{total}}}$$

**Figure 13:**
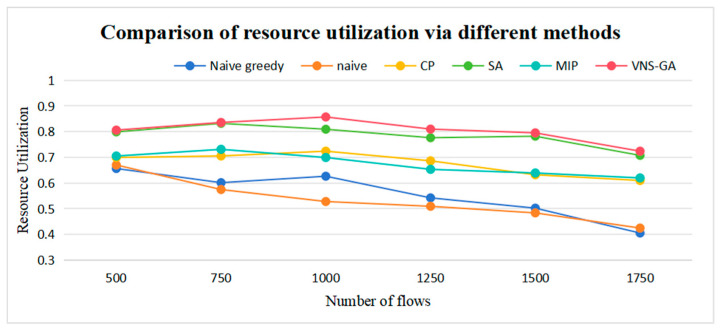
Comparison of resource utilization via different methods.

The higher the resource utilization rate, the more fully the equipment is utilized. Compared to the other three algorithms excluding the VNS-GA and SA, the resource utilization rate of the MIP algorithm increased by an average of 21.4%, 26.85%, and 2.37%, respectively. For the VNS-GA, the resource utilization rate increased by an average of 44.81%, 51.32%, 19%, and 2.57%, respectively, compared to the other four algorithms (excluding MIP). The resource utilization rates are illustrated in Figure 13. The traditional method effectively handles complex constraints. However, it may overlook the impact of certain constraints, leading to unreasonable resource scheduling. In contrast, the MIP method can accurately define and manage multiple constraints to ensure optimal resource allocation. The VNS-GA method achieves a balance within the solution space by dynamically adjusting constraints during the search process, thereby avoiding irrational resource allocation.

#### 4.2.5. Variance of Resource Distribution

The horizontal load balancing is measured by the variance of resource distribution across all queues. The expression for resource distribution variance is shown in Equation (14).

(14) $$\mathrm{Var}(u)=\frac{1}{n}\sum_{i=1}^{n}\left(U_{i}-\bar{U}\right)$$

**Figure 14:**
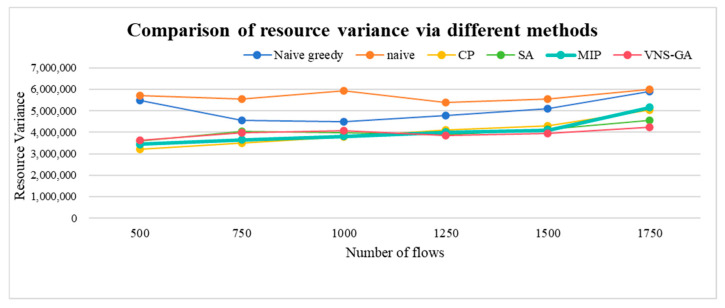
Comparison of resource variance via different methods.

*n* represents the number of resource utilization rates, while *U_i_* denotes the value of each resource utilization rate, indicating the average resource utilization. A lower variance in resource distribution implies a higher level of load balancing, ensuring equitable resource utilization. The proposed MIP algorithm, compared to Naive greedy, Naive, and CP, reduces the resource distribution variances by 12.63%, 21.2%, and 0.23%, respectively. This indicates that within a certain traffic of TSN networks, the MIP algorithm can more effectively utilize resources, achieving superior load balancing and ensuring equitable resource utilization. In comparison to the other four algorithms, the VNS-GA reduces the resource distribution variances by 22.17%, 30.59%, 1.75%, and 2.3%, respectively. This demonstrates the superiority of the VNS-GA in traffic scheduling, as it optimizes resource allocation more effectively, mitigating system load imbalances and enhancing overall system performance and stability. The resource distribution variances are depicted in Figure 14.

#### 4.2.6. Execution Time

**Figure 15:**
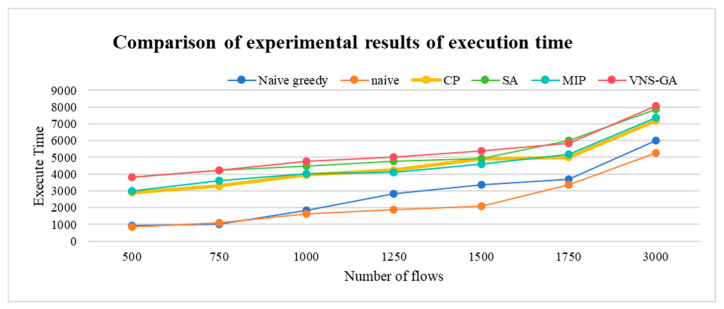
Comparison of experimental results of execution time.

The VNS-GA requires more computational time compared to other algorithms, as it integrates various search strategies, specially VNS and the GA. This diversity aids in a more comprehensive exploration of the solution space, ultimately converging towards superior solutions. The execution time with up to 3000 flows is shown in Figure 15.

#### 4.2.7. Average Delay with Flows of Different Priorities

**Figure 16:**
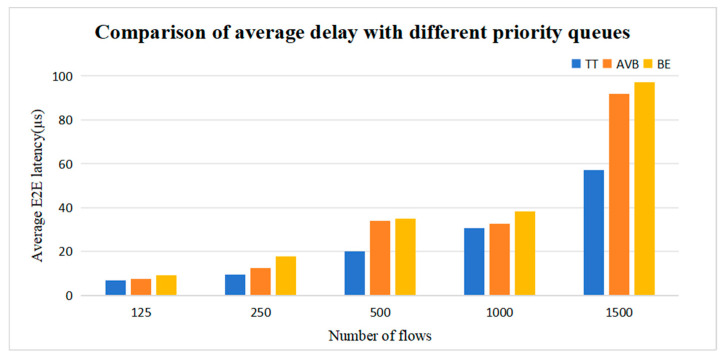
Comparison of average delay with different priority queues.

We compared the transmission delay of Multi-CQF with different priority queues. Considering the varying queue lengths for different priority levels, the queue length for TT flows is set as 250 μs, while for AVB and BE flows, it is set as 125 μs each. The simulation results further demonstrate that the transmission delay for TT flows is significantly less compared to the other two flows, with an average reduction of approximately 30.49% and 37.15%, respectively. This indicates that under the same TSN network conditions, TT flows can achieve faster transmission and reach their destination more quickly, whereas AVB and BE flows exhibit longer transmission delays. These findings underscore the importance of prioritization in TSN network data transmission, particularly in scenarios necessitating swift transmission and timely responsiveness. The results are illustrated in Figure 16.

#### 4.2.8. CQF, CSQF, Multi-CQF

**Figure 17:**
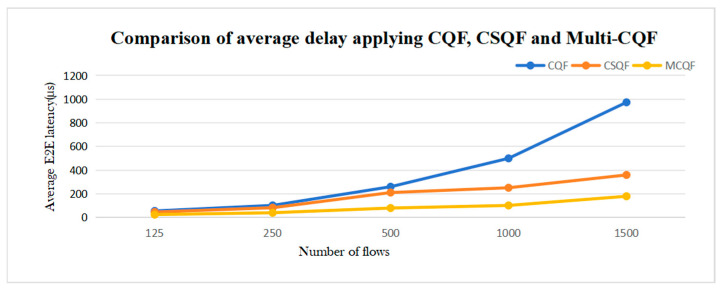
Comparison of average delay applying CQF, CSQF, and Multi-CQF.

We compared the delay performance of CQF, CSQF, and Multi-CQF under different traffic conditions. The simulation results demonstrate that Multi-CQF significantly reduces transmission delay compared to CSQF and CQF, by approximately 50% and 78.2%, respectively. This improvement stems from Multi-CQF’s utilization of a greater number of queues, enabling better traffic management and allocation, thereby enhancing network transmission efficiency. The simulation findings are reflected in Figure 17.

#### 4.2.9. Bi-Objective Lexicographical Statistical Analysis

To conduct a comprehensive comparison of algorithms across various performance criteria, we adopted the bi-objective dictionary-based statistical analysis proposed in [28]. In this method, average end-to-end delay is considered the primary performance metric, while execution time serves as a secondary tie-breaking factor.

This analysis covers all 13 test instances used in our experiments, encompassing both small-scale and large-scale traffic scenarios. For each case, algorithms are first ranked based on their average delay. If two or more algorithms demonstrate statistically equivalent performance in terms of delay, execution time is used as the decisive factor.

Table 4 summarizes the ranks assigned to each heuristic under this dictionary-based scheme. The results show that MIP achieved the best performance in three small-scale instances (125 to 500 flows), validating its effectiveness for smaller problem sizes. Likewise, the VNS-GA consistently outperformed the others in two small-scale (750 to 1000 flows) and seven large-scale (1250 to 3000 flows) scenarios, demonstrating the strength of the proposed method in large-scale optimization problems.

## 5. Conclusions

This paper proposes a TSN network traffic model for Multi-CQF traffic scheduling based on queuing theory, and employs two distinct methods to optimize end-to-end delay for different traffic TSN networks. Queueing theory, serving as a preprocessing step, offers initial solutions to assist in maintaining load balancing at each step of scheduling, thereby enhancing the global load balancing level. MIP continuously explores the feasible solution space through branch-and-bound and linear programming techniques, updating the optimal solution and upper bounds during the search process. However, in large-traffic networks containing numerous nodes and edges, MIP computations may become prohibitively expensive. In such cases, heuristic algorithms may offer a preferable alternative. Within the VNS-GA, genetic operations are employed to preserve and propagate excellent genetic information, introducing new variations to foster diversity and exploration. Subsequently, by altering the local structure of solutions through variable neighborhood search, the surrounding solution space is explored to discover superior scheduling schemes. Compared with other existing schemes, computer simulation demonstrated that MIP reduces delay by approximately 13% on average in small-traffic TSN networks, while the VNS-GA achieves an average delay reduction of approximately 7% on average in large-traffic ones.

Future research on TSN network traffic scheduling will focus on optimizing delay through several key strategies. First, classification algorithms can be employed for various traffic types to ensure efficient resource utilization and maintain the Quality of Service (QoS), accommodating multiple priorities for effective scheduling. Additionally, scheduling compression methods can be explored to mitigate jitter during frame transmission, thereby reducing delay and enhancing stability. The integration of fuzzy knowledge-based algorithms can further decrease computational complexity, improving scheduling efficiency. To validate these approaches, an experimental testbed should be established that simulates real network environments with diverse topologies and traffic scenarios, allowing us to evaluate the performance of the proposed methods in practical settings. This comprehensive testing will provide valuable insights into the feasibility and effectiveness of novel algorithms, supporting the deployment of TSN networks.

## Figures and Tables

**Table 1:** An example of frame properties.

Frame	Period	Transmission Delay	Route
*f*1	500	10	[ES1, SW1] [SW1, SW2] [SW2, ES3]
*f*2	1000	20	[ES1, SW1] [SW1, SW3] [SW3, ES3]
*f*3	500	10	[ES2, SW3] [SW3, ES3]
*f*4	1000	20	[ES2, SW3] [SW3, SW2] [SW2, ES3]

**Table 2:** The parameter settings of the VNS-GA.

Parameters	Parameters Settings
Maximum number of iterations	500
Population size	150
Crossover probability	0.7
Mutation probability	0.05
Neighborhood search iteration count	1500

**Table 3:** The main characteristics of the four existing algorithms.

Method	Advantages	Limitations	Applicability/Notes
CP	Guarantees optimal solution	High computational cost	Suitable for small-scale problems
SA	Good global search ability	Sensitive to parameters	General-purpose
NG	Fast execution; low complexity	May produce suboptimal results	Useful for baseline or fast approximation
NV	Very fast; implementation-friendly	Ignores dynamic constraints	Mainly used for theoretical comparison

**Table 4:** Ranking table of different methods using bi-objective lexicographical statistical analysis.

Flow Size	125	250	500	750	1000	1250	1500	1750	2000	2250	2500	2750	3000
Naive	6	6	6	6	6	6	6	6	6	6	6	6	6
Naive greedy	5	5	5	5	5	5	5	5	5	5	5	5	5
CP	3	4	3	4	4	4	4	4	4	4	4	4	4
SA	4	3	4	2	2	2	2	2	2	2	2	2	2
MIP	1	1	1	3	3	3	3	3	3	3	3	3	3
VNS-GA	2	2	2	1	1	1	1	1	1	1	1	1	1

## Data Availability

Enquiries about data availability should be directed to the authors.

## References

[1] Bhat S.A., Huang N.-F., Sofi I.B., Sultan M. (2022). Agriculture-food supply chain management based on blockchain and IoT: A narrative on enterprise blockchain interoperability. Agriculture.

[2] Xu L.D., He W., Li S. (2014). Internet of things in industries: A survey. IEEE Trans. Ind. Inform..

[3] Khanmohamadi M., Guerrieri M. (2025). Smart Intersections and Connected Autonomous Vehicles for Sustainable Smart Cities: A Brief Review. Sustainability.

[4] Omari Alaoui A., Oumoulylte M., Oubalahcen H., Boutahir M.K., Hessane A., El Allaoui A. (2025). Real-time traffic signal adjustment using YOLOv8 for improved integration of emergency vehicles in smart traffic systems. Signal Image Video Process..

[5] Oza P., Hudson N., Chantem T., Khamfroush H. (2024). Deadline-aware task offloading for vehicular edge computing networks using traffic light data. ACM Trans. Embed. Comput. Syst..

[6] Tomar I., Sreedevi I., Pandey N. (2022). State-of-art review of traffic light synchronization for intelligent vehicles: Current status, challenges, and emerging trends. Electronics.

[7] (2018). IEEE Standard for Local and Metropolitan Area Networks–Bridges and Bridged Networks.

[8] Messenger J.L. (2018). Time-sensitive networking: An introduction. IEEE Commun. Stand. Mag..

[9] Fischer F., Merli D. Security Considerations for IEEE 802.1 time-sensitive networking in converged industrial networks. Proceedings of the 2022 International Conference on Electrical, Computer, Communications and Mechatronics Engineering (ICECCME).

[10] (2020). IEEE Standard for Local and Metropolitan Area Networks–Timing and Synchronization for Time-Sensitive Applications.

[11] IEEE802.1QbvStandard, Std. https://www.ieee802.org/1/pages/802.1bv.html.

[12] IEEE 802.1Qch Standard, Std. https://1.ieee802.org/tsn/802-1qch/.

[13] Finn N. Multiple Cyclic Queuing and Forwarding. https://www.ieee802.org/1/files/public/docs2021/new-finn-multiple-CQF-0921-v02.pdf.

[14] Nasrallah A., Balasubramanian V., Thyagaturu A.S., Reisslein M. (2019). Cyclic queuing and forwarding for large scale deterministic networks: A. survey. arXiv.

[15] Chen M., Geng X., Li Z. (2018). Segment Routing (SR) Based Bounded Latency. Internet Engineering Task Force, Internet-Draft Draft-Chendetnet-sr-Based-Bounded-Latency-00. https://www.ietf.org/proceedings/103/slides/slides-103-detnet-09-sr-based-bounded-latency-00.pdf?__cf_chl_tk=0e9Rg0tGSoYDZjElZrf1tw6cY4sLYr4NZXxOxwSmIaA-1751351240-1.0.1.1-7oXAAIWoiZouNdpZNlOr5muOztoozT9ZYviQiy5VRXA.

[16] Huang Y., Wang S., Huang T., Liu Y. (2022). Cycle-based time-sensitive and deterministic networks: Architecture, challenges, and open issues. IEEE Commun. Mag..

[17] Craciunas S.S., Oliver R.S., Chmelík M., Steiner W. Scheduling real-time communication in IEEE 802.1 Qbv time sensitive Networks. Proceedings of the 24th International Conference on Real-Time Networks and Systems.

[18] Craciunas S.S., Oliver R.S., Steiner W. (2017). Formal scheduling constraints for time-sensitive networks. arXiv.

[19] Oliver R.S., Craciunas S.S., Steiner W. (2018). IEEE 802.1 Qbv gate control list synthesis using array theory encoding. Proceedings of the 2018 IEEE Real-Time and Embedded Technology and Applications Symposium (RTAS).

[20] (2018). Ultra-Low Latency (ULL) Networks: The IEEE TSN and IETF DetNet Standards and Related 5G ULL Research. Proc. IEEE Commun. Surveys Tutor..

[21] Dürr F., Nayak N.G. (2016). No-wait packet scheduling for IEEE time-sensitive networks (TSN). Proceedings of the 24th International Conference on Real-Time Networks and Systems.

[22] Gao J., Zhu X., Bai K., Zhang R. (2022). New controllable processing time scheduling with subcontracting strategy for no-wait job shop problem. Int. J. Prod. Res..

[23] Wang X., Yao H., Mai T., Guo S., Liu Y. (2023). Reinforcement Learning-Based Particle Swarm Optimization for End-to-End Traffic Scheduling in TSN-5G Networks. IEEE/ACM Trans. Netw..

[24] Yan J., Quan W., Jiang X., Sun Z. (2020). Injection time planning: Making CQF practical in time-sensitive networking. Proceedings of the IEEE INFOCOM 2020-IEEE Conference on Computer Communications.

[25] Zhang Y., Xu Q., Xu L., Chen C., Guan X. (2022). Efficient flow scheduling for industrial time-sensitive networking: A divisibility theory-based method. IEEE Trans. Ind. Inform..

[26] Quan W., Yan J., Jiang X., Sun Z. (2020). On-line traffic scheduling optimization in IEEE 802.1 Qch based time-sensitive networks. Proceedings of the 2020 IEEE 22nd International Conference on High Performance Computing and Communications; IEEE 18th International Conference on Smart City; IEEE 6th International Conference on Data Science and Systems (HPCC/SmartCity/DSS).

[27] Alexandris K., Pop P., Wang T. (2022). Configuration and evaluation of multi-CQF shapers in IEEE 802.1 time-sensitive networking (TSN). IEEE Access.

[28] Carvalho I.A. (2019). On the statistical evaluation of algorithmic’s computational experimentation with infeasible solutions. Inf. Process. Lett..

[29] IEEE 802.1 Working Group (2021). IEC/IEEE 60802 TSN Profile for Industrial Automation. https://1.ieee802.org/tsn/iec-ieee-60802/.

